# Remineralising effect of 45S5 bioactive glass on artificial caries in dentine

**DOI:** 10.1186/s12903-020-1038-4

**Published:** 2020-02-11

**Authors:** Qiong Wu, May Lei Mei, Xin Wu, Shuya Shi, Yuting Xu, Chun Hung Chu, Yaming Chen

**Affiliations:** 1 Jiangsu Key Laboratory of Oral Diseases, Nanjing Medical University, Nanjing, China; 2 Department of Polyclinics, Affiliated Hospital of Stomatology, Nanjing Medical University, Nanjing, China; 3 Faculty of Dentistry, University of Otago, Dunedin, 9054 New Zealand; 4 Department of Stomatology, Nanjing First Hospital, Nanjing Medical University, Nanjing, China; 5 Department of Stomatology, Affiliated hospital of Jiangnan University, Wuxi, China; 6 Stomatological Hospital Affiliated to Soochow University, Suzhou, China; 7 Faculty of Dentistry, The University of Hong Kong, Hong Kong, China

**Keywords:** Artificial caries, Dentine, Remineralisation, Bioactive glass, CPP-ACP

## Abstract

**Background:**

This study investigated the remineralisation effect of bioactive glass on artificial dentine caries.

**Methods:**

Dentine disks with artificial caries were treated with bioactive glass (group BAG), casein phosphopeptide–amorphous calcium phosphate (CPP-ACP) (group CPP-ACP), sodium fluoride glycerol (group F) or deionized water (group W). All disks were subjected to pH cycling for 28 days subsequently. The topography, microhardness and remineralisation depth of the dentine carious lesion were assessed by atomic force microscopy (AFM), microhardness testing and confocal laser scanning microscope (CLSM), respectively.

**Results:**

AFM images indicated mineral depositions on the surface of the carious lesion in group BAG. The changes of Vickers hardness number (ΔVHN, mean ± SD) after pH cycling were 9.67 ± 3.60, 6.06 ± 3.83, 5.00 ± 2.19 and − 1.90 ± 2.09 (*p* < 0.001) in group BAG, group CPP-ACP, group F and group W, respectively. The remineralisation depth (mean ± SD) of the carious lesion in group BAG, group CPP-ACP, group F and group W were 165 ± 11 μm, 111 ± 11 μm, 75 ± 6 μm and 0 μm (*p* < 0.001), respectively.

**Conclusion:**

Bioactive glass possessed a promising remineralisation effect on artificial dentine caries and could be a therapeutic choice for caries management.

## Background

Dental caries (tooth decay) is one of the most prevalent chronic disease [1]. Dentine caries refers to the situation in which caries has progressed into dentine and caused significant lesion depth, it can progress rapidly since dentine is a porous organic-inorganic composite material. The traditional management of dentine caries has focused primarily on treatment via the excision of diseased tissues and the subsequent restoration of the defect [2]. The primary goal of contemporary mineral invasive dentistry is to respect tooth structure, retaining viable and biologically repairable tissues to maintain tooth vitality. Therefore, retaining demineralised dentine which has no bacteria invasion and restored it with bioactive materials which has remineralization capability is the trend of caries treatment. This procedure can not only prevent further bacterial infection, but also preserve dental hard tissues as much as possible, which is beneficial to protect dental pulp tissues, and increase the retention ability and resistance performance of restoration materials [3]. Bioactive materials play an important role in treatment of the partial removal of caries.

Bioactive materials are therefore been introduced since whey will be intended to interact in some positive way with the oral environment. 45S5 bioactive glass (BAG) was initially introduced in 1970s, it is a glass in Na_2_O-CaO-SiO_2_-P_2_O_5_ system, high in calcium content [4]. It was found to be able to bond with bone rapidly and strongly, it stimulates bone growth away from the bone–implant interface [5]. The mechanism for bone bonding is attributed to a hydroxycarbonate apatite (HCA) layer on the surface of the glass, following initial glass dissolution. BAG has been introduced into dentistry to treat dentine hypersensitivity in 2004 [6]. In vitro studies showed that the BAGs particles can adhere to dentine and form an HCA layer which is similar in composition to dentine, therefore block the dentinal tubules [7]. This indicates that BAG seems to work by stimulating mineralization (calcium phosphate deposition over the dentine tubules) [8, 9].

Apart from treating dentine hypersensitivity, BAG has been used in different areas in dentistry. A.S. Bakry’s studies showed that BAG can be used to treat enamel leukoplakia caused by orthodontic treatment and as a temporary filling material for remineralization [10, 11]. BAG also can be used as an auxiliary material for tooth bleaching to prevent/repair the damage caused by enamel bleaching agent [12]. Research shows that a novel BAG has been developed to as a viable alternative to adhesive removal with a TC bur [9]. A combined dentine pre-treatment using BAG followed by polyacrylic acid may increase the bond strength and maintain it stable over time [13]. Increasing BAG filler content in pit and fissure sealants may prevent secondary caries at enamel edge [14]. However, the effect and mechanisms of BAG on dentine caries is still unclear.

It had also been reported that several other materials could remineralize dentin, including casein phosphopeptide-amorphous calcium phosphate (CPP-ACP) and fluoride compounds [1, 15, 16]. CPP-ACP enhances remineralization by stabilizing calcium phosphate so that high concentrations of calcium ions and phosphate ions exist in the solution. Fluoride has been shown to enhance the remineralisation of caries [17]. Fluoride is mainly combined with supersaturated calcium and phosphorus ions to further promote the deposition of calcium and phosphorus, forming new antacid fluorapatite crystals and realizing remineralization. These studies have proclaimed sufficient observations to prove the formation of mineral depositions on dentin surface after treatment. In this study, CPP-ACP and sodium fluoride are used as positive controls, pH-cycling model was used to simulate the dynamic variation in mineral saturation and the pH altering with the natural caries process, which refers to in vitro experimental protocols including exposure of dentin to combinations of demineralization and remineralization. The null hypothesis of the study is that BAG does not have reminerlisation effect on artificial dentine caries.

## Methods

### Dentine disks preparation

**Fig. 1:**
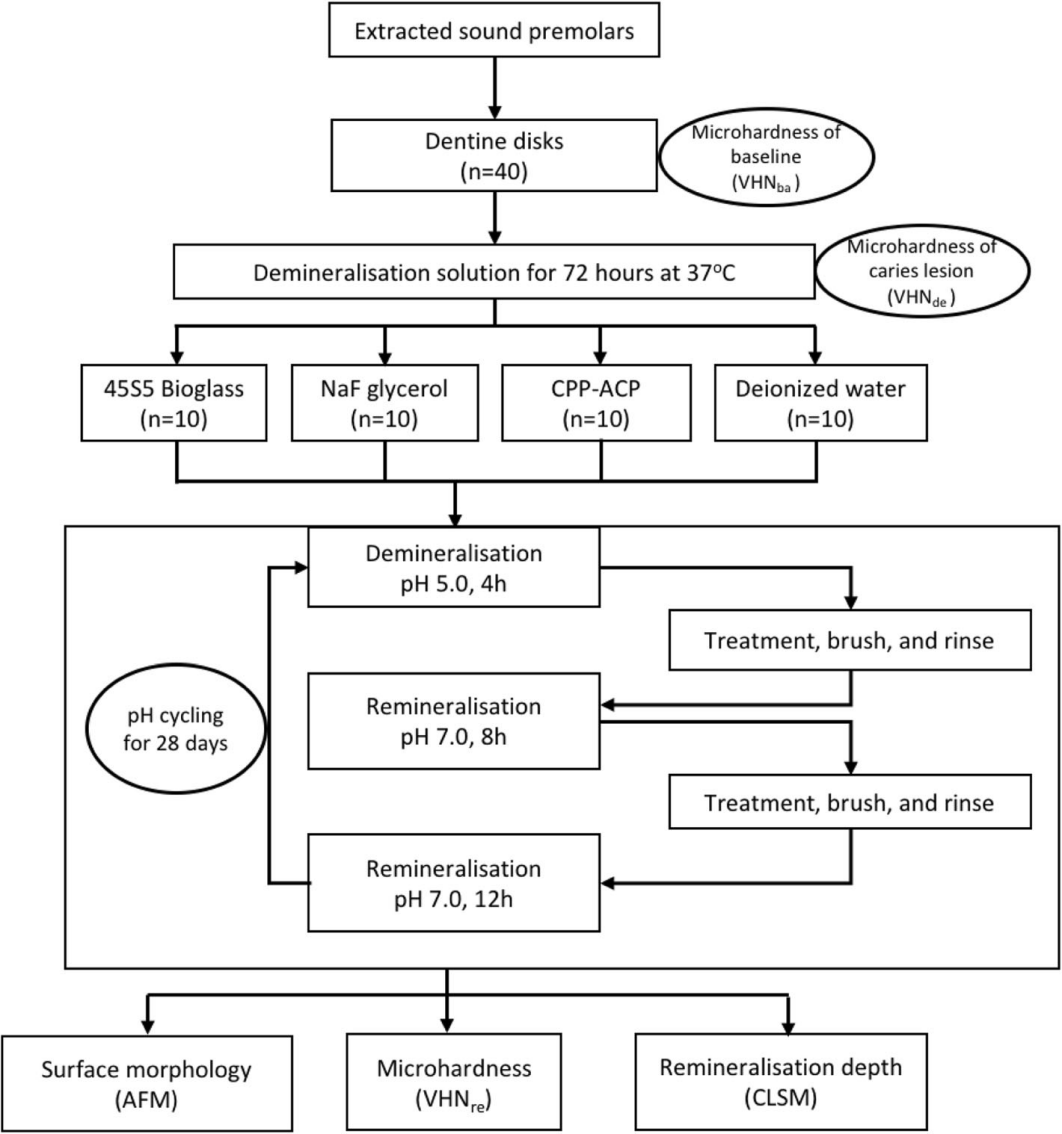
Flowchart of experimental design

Ethical approval was obtained from Ethics Committee of the School and Hospital of Stomatology, Nanjing Medical University (2019–284). This study was conducted in full accordance with the Declaration of Helsinki of the World Medical Association. All participants received dental treatment at the Hospital of Stomatology of Nanjing Medical University and provided written informed consent. The written consents were obtained from the parents/guardians of the teenagers who were under 16 years of age. Forty human premolars extracted within one month for orthodontic reasons were collected and stored in deionized water containing 0.1% thymol at 4 °C prior to the experiment. Crowns with caries, restorations, or fractures were abandoned. The flow chart in Fig. 1 summarises the protocol of this study.

Forty dentine disks with a thickness of 1.0 mm, perpendicular to the long axis of the tooth above the cemento-enamel junction, were prepared by low-speed water cooled diamond saw (Isomet, Buehler Ltd., Lake Bluff, IL, USA). All disks were free of coronal enamel or pulpal exposures. A standard smear layer was created on the coronal side of the dentin surface using silicon carbide papers of 600-grit, 800-grit, 1200-grit and ultrasonically washed in deionized water 3 times each for 60s, while the opposite sides were coated with acid-resistant nail polish.

### Demineralisation and remineralisation solutions

The demineralisation solution contains was 0.05 M acetic acid containing 2.2 mM CaCl_2_·2H_2_O (Shanghai Ling Feng Chemical Reagents Co., Ltd.,) and 2.20 mM KH_2_PO_4_ (Shanghai Ling Feng Chemical Reagents Co., Ltd.,) and was adjusted to pH 5.0.

The remineralisation solution contained 1.5 mM CaCl_2_·2H_2_O, 0.90 mM KH_2_PO_4_ and 130 mM KCl (Shanghai Ling Feng Chemical Reagents Co., Ltd.,), and was adjusted to pH 7.0. Both of them were freshly prepared [18].

### Preparation of artificial lesions

All disks were immersed into deminerlisation solution for 72 h at 37 °C. The surface hardness of the disks was characterized by Vicks microhardness number (VHN).

### Experimental procedure

The demineralized dentine disks were randomly assigned into four groups (*n* = 10). The treatments were applied twice a day using electric toothbrush (Colgate 360°, Colgate- Palmolive Co.), the disks were rinsed thoroughly after brushing to mimic the real situation.

Group 1: 0.075 g/mL BAG paste (Actimins Paste, Datsing Bio-Tech Co. Ltd., Beijing, China), (Na_2_O_2_ 4.5 wt%, CaO_2_ 4.5 wt%, P_2_O_5_ 6.0 wt%, SiO_2_ 45 wt%).

Group 2: Sodium fluoride and Glycerin Paste (75% sodium fluoride and 25% Glycerol).

Group 3: 10% CPP-ACP (Recaldent™, Japan GC Co.,Ltd) (CPP–ACP:10%; Ca content: 13 mg/g; P content: 5.6 mg/g).

Group 4: Deionized water.

All disks were subjected to 28 days’ pH cycles, which consisted of 4 h demineralization solution followed by a 20 h remineralisation solution. Each disk was placed in a 15 mL container. All the solutions were freshly made prior to use. All the disks were collected for testing after pH cycling.

### Surface roughness test

Three disks from each group embedded in epoxy resin were imaged using an atomic force microscope (AFM; CSPM 5000, Ben Yuan Ltd., Beijing, China) in order to analyze surface morphology changes. The dentine disks were polished with silicon carbide paper (2000 grit), then 1.0, 0.3 and 0.05 μm diamond mask alumina suspensions sequentially, followed by ultrasonically cleaning in deionized water for 15 min to remove the residues [19].

Topographical images of the surface were performed in the tapping mode using a silicon nitride scanning probe in admosphere, in which the probe periodically touches the sample surface, producing higher quality images [15]. Each dentine disk was observed in 4 different sites and obtained three-dimensional images of the dentin surface. In each image, a field of view at 50 μm × 50 μm scan size, 1.5 Hz scan rate and a resolution of 512 by 512 pixels were employed on the entire surface.

### Surface microhardness test

Seven disks from each group were randomly selected to measure the microhardness respectively of baseline (VHN_ba_), before pH cycling (VHN_de_) and after pH cycling (VHN_re_). The microhardness value of each disk was measured with a Vickers indenter on a Hardness Tester (DHV-1000, Shangcai testermachine Co., LTD, China).

Indentations were made with a Vickers diamond indenter of three widely similarly positioned locations. The indentations with loads of 0.98 N and time for 15 s were considered to be suitable for dentin measurement of the long and short indentation diagonals and resulted in minimum surface damage. As the apexes of the diagonals were estimated on the surface, Vickers number could be converted by the size of the indentation. Three values were averaged to produce one hardness value for each specimen. The change in Vickers hardness number (ΔVHN) was determined as the difference of the caries lesion before and after pH cycling (ΔVHN = VHN_re_ - VHN_de_).

### Confocal laser scanning microscopy (CLSM)

The disks from microhardness study were cut to thin sections with thickness of 500 μm along the treatment surface, and then stained with a freshly prepared 0.1% Rhodamine B solution (Aldrich Chem. Co., Milwaukee, WI, USA) for 1 h, and rinsed for 3 times with deionized water. Samples were analyzed with a confocal laser scanning microscopy (CLSM, CarlZeiss LSM 710, Carl Zeiss, Inc., Germany). The reflection imaging was performed using the laser. Standard settings for contrast, brightness and laser power were used for all images. The remineralisation depths (H) were quantitatively analyzed with an image-analysis system (Image Pro-Plus, 6.0).

### Statistical analysis

All of the data were assessed for a normal distribution using the Shapiro–Wilk test for normality (*p* > 0.05). A one-way ANOVA was used to compare the VHN and remineralisation depth across the four treatment groups, followed by LSD multiple comparison was used to compare among groups. All of the analyses were conducted using IBM SPSS Version 2.0 software (IBM Corporation, Armonk, New York, USA). The cut-off level for significance was taken as 5% for all of the analyses.

## Results

**Fig. 2:**
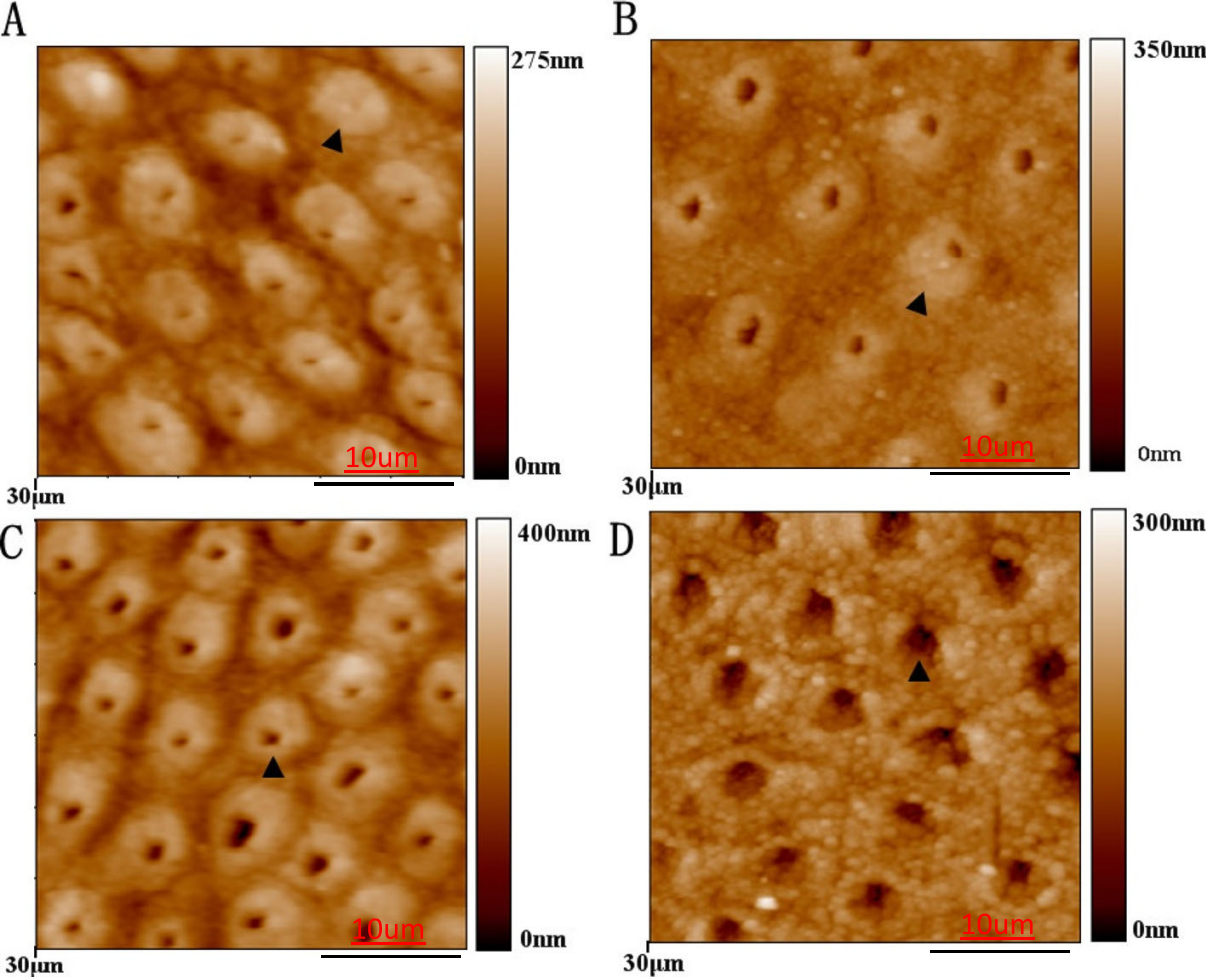
AFM micrographs in the tapping mode of specimen surfaces after 28-day treatment by bioactive glass **a**, sodium fluoride glycerin **b**, CPP-ACP **c** and deionized water **d**

Figure 2 showed the surfaces of the dentine disks after treatments and pH cycling. We observed that dentine collagen fibres were not exposed on the relatively smooth surface of the BAG, fluoride and CPP-ACP treated dentine (Fig. 2 a, 12B and 2C). In particular, parcipatation on the peritubular dentine, and little space remained in both inter-tubular and intra-tubular areas. Figure 2 d is the negative control which received water, enlarged dentinal tubulars when comparing with other groups, indicating partial demineralisation.

The means and standard deviations of VHN of the dentine of 4 groups of the baseline, demineralized and after pH cycling are summarized in Table 1. Group BAG, Group CPP-ACP and Group F showed higher VHN when comparing Group W after 28 days pH cycling (*p* = 0.020). There was no significant difference in VHN among different groups in the baseline (*p* = 0.919), as well as after 72 h demineralization (*p* = 0.290). Group BAG and Group CPP-ACP presented larger ΔVHN when comparing with Group F (*p* < 0.001).

**Table 1:** Mean VHN and SD of dentin surface in sound dentine, after demineralisation and after pH cycling. VHN, Vickers microhardness numbers

Group (*n* = 7 each)	VHN_ba_ Mean ± SD	VHN_de_ Mean ± SD	VHN_re_ Mean ± SD	ΔVHN (VHN_re_ ­- VHN_de_) Mean ± SD
Group BAG	60.44 ± 6.41	13.05 ± 2.90	22.72 ± 3.91^a^	9.67 ± 3.60^a^
Group CPP-ACP	60.67 ± 3.53	15.60 ± 4.62	21.66 ± 3.92^a^	6.06 ± 3.83^a^
Group F	60.07 ± 9.95	13.35 ± 4.26	18.35 ± 5.62^a^	5.00 ± 2.19^b^
Group W	58.36 ± 2.82	16.82 ± 3.31	14.92 ± 3.23^b^	− 1.90 ± 2.09^c^
*p* value	0.919	0.290	0.020	< 0.001
LSD comparison	N/A	N/A	a > b	a > b > c

**Fig. 3:**
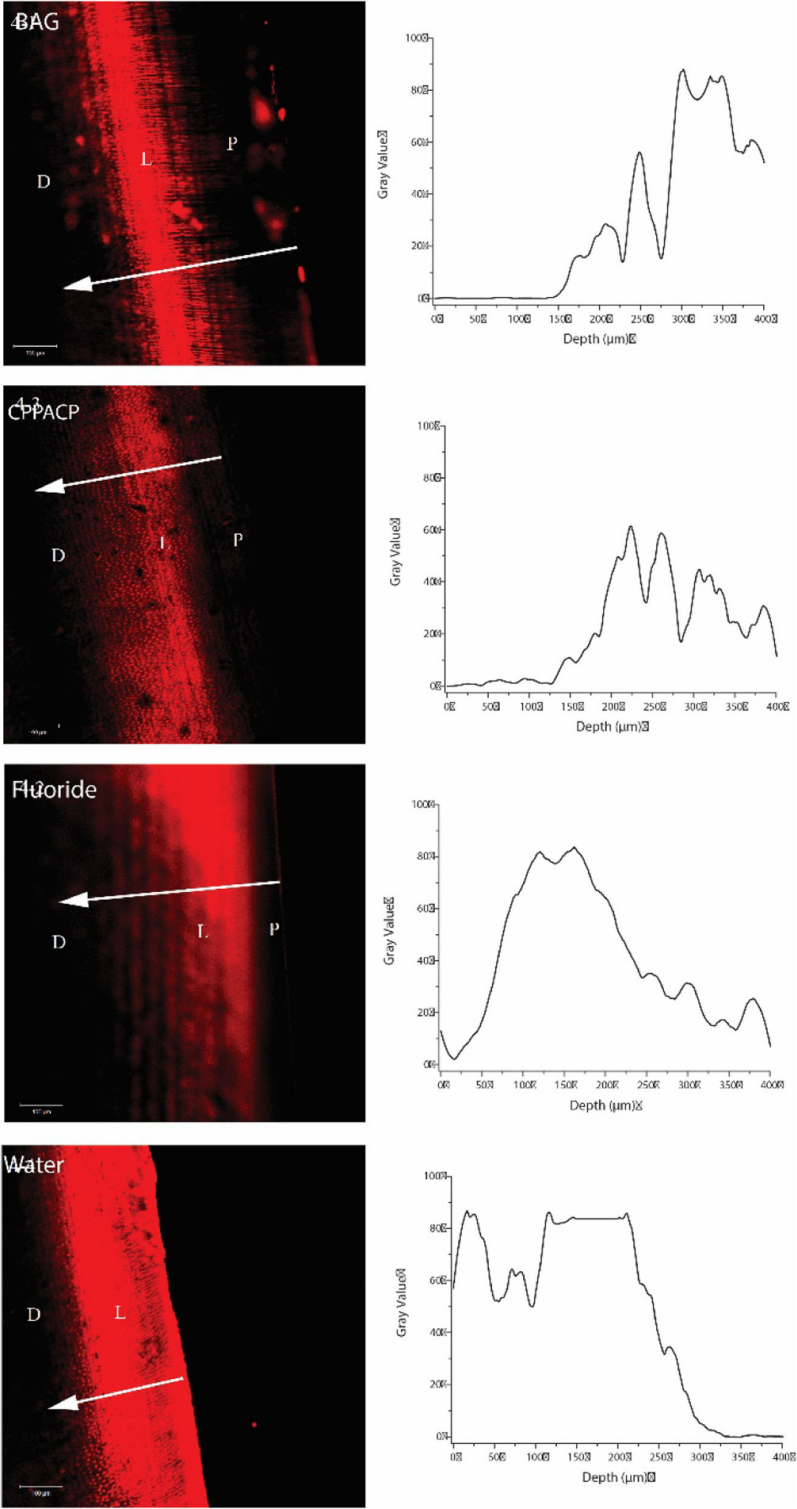
Confocal Laser Scanning Microscopy representative image of artificial dentin caries treated by bioactive glass (4–1), sodium fluoride glycerin (4–2), CPP-ACP (4–3) and deionized water (4–4). (L, lesion; D, sound dentin; P, precipitation band)

CLSM observation showed a red fluorescent band representing caries lesion. The remineralization is evidenced by the decrease in fluorescence on the superficial layer of the lesion (Fig. 3). The precipitation band was wider in Group BAG when compared to the fluoride treated and the control group. Correspondingly, Table 2 show the depth of remineralisation zone after 28 days pH cycling in the four experimental groups. The depth of remineralisation zone of Group BAG is 165.40 ± 11.09 μm, which is significantly higher (*p* < 0.001) than those in other groups, demonstrating a promising capability in remineralising dentine caries. Combined with the CLSM images, BAG promoted mineral deposition on the superficial layer of the lesion.

**Table 2:** The depth of dentine remineralisation zone in 4 experimental groups (n = 7)

Group	Depth (μm)
Group BAG	165.40 ± 11.09^a^
Group CPP-ACP	110.86 ± 10.55^b^
Group F	75.18 ± 6.47^c^
Group Water	0
*p value*	< 0.001
LSD comparison	a > b > c

## Discussion

This study investigated the remineralization effect of BAG on artificial dentine caries. It provides useful information about the microstructure changes in dentine caries after BAG application. According to the result of the study, the null hypothesis was rejected. BAG showed a promising remineralization effect on artificial dentine caries with increasing microhardness by forming a remineralization zone on the lesion surface. Hardness testing is an indirect method of tracking changes in the mineral content of dentine, and several microhardness studies on dentine in arrested carious lesions have been published [20, 21]. A limitation of the study is the chemical system used is the lack of biological component, in which antimicrobial of the treatment could be underestimated. A biological model can be employed in the next step to assess antimicrobial effect. In addition, the results cannot be extrapolated to the in vivo situation and caution should be exercised in their interpretation. In AFM study, the specimens require high quality polished surface. Polishing teeth could remove some attachments on the surface, but according to the AFM results, the BAG mainly embedded into dentin tubules to form deposits.

Two perspectives have been focus to achieve dentine caries remineralisation: coating nucleation templates on demineralized dentin or creating a local environment with high calcium and phosphorus concentration [22–24]. The process of remineralising dentine caries using BAG includes exchanging ions (Na^+^, Ca^2+^, PO_4_^3−^, F^−^) in the silicate network of BAG with the surrounding oral liquid to supersaturate the ions in the fluid, which are then reprecipitated on the silicate network of BAG in the tissue [25]. BAG can make materials and tissues bond tightly, which is conducive to promoting the remineralization of calcium phosphate on the surface of teeth in vivo [26]. It can promote the formation of stable crystalline hydroxyapatite crystals on the surface of demineralized teeth in salivary environment, thus promoting dentine caries remineralisation. In current study, a very fine BAG powder (Actimins Paste, Datsing Bio-Tech Co. Ltd., Beijing, China) with the maximum grain size is less than 90 nm was used [27]. Small size particles facilitate the penetration into dentine caries, they also provide large surface area for reaction.

It had been demonstrated that dentine remineralisation occurs neither by spontaneous precipitation nor by nucleation of mineral on the organic matrix but by growth of residual crystals in the lesions [28]. And as it has been discovered by researchers that remineralization was possible even at a high degree of initial mineral loss, where it might have been considered that the caries process had happened [29]. It is advantageous to save softened but not the bacterial invasion demineralization dentin, which is consistent with minimum damage strategy for dentin caries treatment. Therefore, various active researches are currently being carried out to seal the exposed dentin tubules with some effective materials and improve the bonding at the dentin interface so as to repair demineralized dentin through remineralization.

Fluoride ions promote the formation of fluorapatite in enamel in the presence of calcium and phosphate ions produced during enamel demineralization by plaque bacterial organic acids. This is now believed to be the major mechanism of fluoride ion’s action in preventing enamel demineralization [30, 31]. It was documented that the anti-cariogenic effects of fluoride mainly through two principal mechanisms: inhibiting demineralization when fluoride is present on crystal surface during an acid challenge; and enhancing remineralization by forming a low soluble substance similar to acid-resistant mineral fluorapatite covering the crystal surface [9, 32]. Some scholars have also found that when the demineralized dentin does not contain hydroxyapatite, no new hydroxyapatite crystals will nucleate after immersion in the remineralized solution. Research has shown that fluoride has limited ability to remineralize dentin when the residual crystals of the lesion are insufficient [33]. CPP-ACP, which has been consider to promote remineralization of the carious lesions by maintaining a supersaturated state of enamel mineral, plays a key role in the biomineralization of dentin [15, 34]. It has also been suggested that CPP-ACP has a multifactorial anticariogenic mechanism. A vitro study showed that the presence of CPP-ACP prevents demineralization of dentin surface and promotes the remineralization of artificial caries-like dentin lesions.

In current study, the treatments were applied on the dentine disks via brushing by an electric toothbrush for 2 min, to mimic the real situation. The mineral was shown to deposit on the surface of the caries lesion in all treatment groups due to the AFM results (Fig. 2), which indicate that daily brush will not remove the deposit. We found that BAG group has the largest remineralisation depth when compared to other groups (Table 2). Ten Cate summarized the factors that enhance the remineralisation of deep lesions, and proposed that calcium may be rate limiting in remineralization [35]. Pronounced bonding capacity to tooth structure of BAG can be a major reason for this improved remineralisation effect. Based on results of this in vitro study, we believe that BAG inhibits the demineralization and/or promotes the remineralisation of artificial dentine caries under dynamic pH-cycling conditions. BAG has potential to a promising alternative to fluoride in the treatment of caries.

## Conclusions

BAG possessed a promising remineralisation effect on artificial dentine caries and could be a therapeutic choice for caries management.

## Data Availability

The datasets used and/or analysed during the current study available from the corresponding author on reasonable request.

## Abbreviations

AFM: Atomic force microscopy
BAG: Bioactive glass
CLSM: Confocal laser scanning microscope
CPP-ACP: Casein phosphopeptide–amorphous calcium phosphate
VHN: Vickers hardness number
